# Outcome of Alcohol Dependence: The Role of Continued Care

**DOI:** 10.4103/0970-0218.51226

**Published:** 2009-04

**Authors:** Pratima Murthy, Prabhat Chand, MG Harish, K Thennarasu, S Prathima, Karappuchamy, N Janakiramiah

**Affiliations:** Department of Psychiatry, National Institute of Mental Health and Neurosciences, Bangalore - 560 029, India; 1 Department of Biostatistics, National Institute of Mental Health and Neurosciences, Bangalore - 560 029, India

**Keywords:** Alcohol, community, outcome

## Abstract

**Aims:**

This study attempted to determine the effects of continued care on subjects with alcohol dependence.

**Materials and Methods:**

Study patients were recruited from a slum in Bangalore. The control group comprised individuals from a lower socio-economic status. Both groups received identical treatment from a specialised de-addiction facility. The study group also received weekly continued care in the community, either at a clinic located within the slum or through home visits. Those patients without stable jobs were referred for employment. The control group was given routine hospital follow-up visits. Both groups were evaluated on the Alcohol Problem Questionnaire and quantity/frequency of drinking at baseline and every 3 month interval for one year after discharge.

**Results:**

Both groups showed improvement in terms of reduction of drinking at 3 months, with the study group showing a 64% improvement with respect to the number of non drinking days and the control group showed a 50% improvement. However, at 6 months, 9 months, and 12 months, the study group continued to maintain these gains while the control group showed a downward slide (differences significant at *P*< 0.05). At the end of 12 months, the study group maintained a 53% improvement with respect to the number of non drinking days as compared with baseline, while the control group had an improvement of only 28%.

**Conclusions:**

Follow-up support and continued care appear to significantly improve longer-term recovery in alcohol dependents.

## Introduction

The treatment outcome of alcohol dependence has always been a matter of concern to both researchers in the area as well as policy planners. Most studies focus on the impact of the first intervention, whether it is brief versus extended,(1) in-patient or out-patient,(2) or the modality of treatment.(3) It is increasingly being identified that short-term interventions have little long-term impact.(4–6) A few recent studies have focused on the influence of continuing social and community support for long-term recovery.(5,7) Alcohol dependence, like other chronic illnesses such as diabetes and hypertension, is well known to have a chronic, relapsing course.(8) For such conditions, it is important to look at issues beyond just the initial setting or type of treatment to whether follow-up support and continued care have any role in maintaining long-term improvement.

This study examined the effects of follow-up and continued care in patients with alcohol dependence from an urban slum.

## Materials and Methods

The study attempted to compare the effects of continued care in follow-up in two groups of patients who received similar hospital-based treatment for alcohol dependence. We examined drinking outcomes, employment patterns, and family functioning in both groups at 3 months, 6 months, 9 months, and 12 months.

## Methodology

This study was carried out in the de-addiction service of the National Institute of Mental Health and Neurosciences, Bangalore, India. Patients were included in the study after written informed consent was obtained. The study group comprised patients from an urban slum referred for treatment for alcohol dependence. Inclusion criteria were ICD-10(9) criteria for alcohol dependence, history of having held employment sometime during their life, and living with family. Patients with a history of serious criminal activity or major physical or psychiatric illness were excluded from the study. Patients in the control group consisted of age-matched patients of similar socio-economic background but not residing in the same slum.

After obtaining written informed consent, both groups were assessed at baseline for socio-demographic details, quantity and frequency of substance use, and related dysfunction. They were also given the Alcohol Problem Questionnaire.(10) Both groups received comprehensive care, predominantly in-patient, which included detoxification and individual, group, and family counseling. The average in-patient stay was 21-28 days.

Following discharge, the study group received continued care through weekly visits to the community by trained counselors. Follow-up visits were carried out in the community clinic within the slum, and a home visit was subsequently made if the patient did not attend the community clinic. The follow-up visit focused on simple relapse prevention messages and providing support to the patient and families. Patients were encouraged to resume work. For those unable to find work immediately, supported employment at minimum wage was offered in the rehabilitation section of the hospital. Patients in supported employment were also counseled regarding relapse prevention.

Patients in the control group were given routine fortnightly follow-up visits at the hospital out-patient clinic. Those who did not show up for routine visits were sent a reminder letter.

Follow-up evaluation was carried out at 3-month intervals following discharge for a total period of 12 months (3^rd^, 6^th^, 9^th^, and end of 1 year). At baseline and at the follow-up visits, the information obtained was corroborated by both the patient and family member.

Data was analyzed using Student's t-test, and paired t-test to compare the study and control groups at baseline and at 3, 6, 9, and 12 months following discharge.

## Results

A total of 99 patients were included in the study, with 50 patients in the study group and 49 patients in the control group. The study group consisted of 48 males and 2 females, while the control group consisted entirely of males. A total of 45 patients in the study group and all 49 patients in the control group opted for the in-patient treatment program.

Both groups were comparable with respect to age, marital status, education, professional skill, and family history of alcohol dependence. The control group was slightly better educated (1 year more on average compared to the study group) [Table 1]. The groups were comparable on years of alcohol use i.e., mean 13.54 years (SD 7.96 years) and 12.45 years (SD 8.06 years) in the study and control group, respectively.

**Table 1:** Socio-demographic characteristics of patients in the study and control groups (baseline)

Socio-demographic variables	Study group (n=50)	Control group (n=49)
Mean age (yrs) ± SD	36.68 ± 7.49	35.02 ± 6.80
Education (yrs) ± SD	4.1 4 ±3.46	5.33 ± 3.72
Professionally skilled (%)	39.00 (78)	41.0 (83.67)
Married (%)	37 (74)	38 (77.6)
Family history of alcohol dependence (%)	42(84)	41 (82)

At baseline, with respect to the previous month, patients in the control group reported significantly more mean days of alcohol use 28.84 days (SD 3.91 days) compared with the study group 25.96 days (SD 6.05 days), (*P* = 0.006).

At baseline, the study group was significantly worse off in terms of work with fewer paid working days in the previous month (7.34 days [SD 8.18 days] in the study group compared with 11.88 days [SD 11.4 days] in the control group [*P*=0.026]).

Follow-up information was obtained at 3 months (90% in the study group and 88% in the control group), 6 months (88% in the study group and 88% in the control group), 9 months (76% in the study group and 88% in the control group), and 12 months (82% in the study group and 88% in the control group).

**Figure 1:**
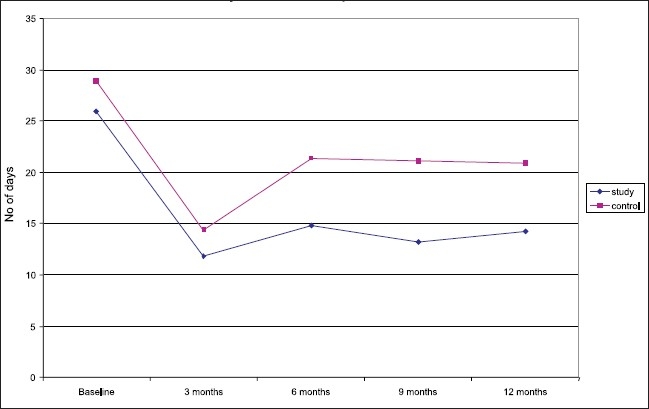
Days of alcohol use in the past month: Comparison of the study group at baseline and follow-up

Both the groups showed improvement in terms of reduction of drinking days at 3 months (a 64% improvement with respect to the number of non drinking days in the study group and 50% in the control group). The study group continued to maintain a 53 to 59% improvement compared with baseline with respect to the number of non drinking days, whereas in the control group, the initial gains declined to between 25 to 28% at follow-up. The reduction in the number of drinking days in the study group was significantly more than the control group at follow-up at 6, 9, and 12 months, respectively (*P* <0.05) [Table 2], [Figure 1].

**Table 2:** Number of days of alcohol use (over the past one month) (days ± SD)

Months	Study group	Control group	t-value	*P* value
Baseline	25.96±6.05	28.84±3.91	0.006 (df=84.1)	0.006
3^rd^ month	11.80±13.84	14.37±14.40	0.851 (df=85)	0.397
6^th^ month	14.82±13.68	21.37±13.45	2.252 (df=85)	0.027
9^th^ month	13.24±13.57	21.05±13.45	2.246 (df=78)	0.012
12^th^ month	14.24±13.69	20.93±13.60	2.246 (df=82)	0.027

None of the patients attended AA groups despite regular advice to do so. At the end of 1 year, the study group showed a statistically significant greater improvement in the areas of marital and employment problems (*P*<0.05) and in areas of problem with children (*P*<0.01) in comparison with the control group [Table 3]. While both groups showed improvements in the number of days worked in the previous month during the follow-up phase, the improvements in the control group tended to fall at the 9^th^ month and 12^th^ month, respectively, and at the 12^th^ month the improvement in the study group was more significant (*P*<0.05).

**Table 3:** Comparison on the alcohol problem questionnaire

Areas	Intake (baseline)		6^th^ month		12^th^ month	
			
	Study group	Control group	Study group	Control group	Study group	Control group
Common problems	9.1	10.6	4.2	3.7 (p=0.05)	3.8	5.9
Marital problems	3.8	3.8	1.3	1.7	1.1	2.8(*P*=0.01)
Problems with children	1.7	1.4	0.8	0.7	0.5	1.2(*P*=0.003)
Employment problems	3.7	3.9	1.5	1.6	0.9	2.6(*P*=0.01)
Total score	18.3	19.7	7.8	7.7	6.3	12.5 (*P*=0.06)

Although the study group had significantly more number of days with employment problems in the previous month at baseline, this difference disappeared during follow-up with the study group catching up in terms of an improved work performance. At the end of 1 year of follow-up, the study group had more earnings per month than that of the control group (although not significant). There was a consistent increase in the amount of earnings per month in the study group where in the control group there was a decline after the initial increase in income in the first 6 months [Table 4].

**Table 4:** Number of days of paid employment in the last 30 days (1 year follow-up)

Months	Study group	Control group	t-value	Significance *P* value
Baseline	7.32±8.00	11.98±11.50	2.344 (df=97)	0.021*
3^rd^ month	17.75±9.76	18.98±9.07	0.198 (df=85)	0.545
6^th^ month	15.14±10.71	18.40±8.49	1.571 (df=85)	0.120
9^th^ month	15.95±10.66	17.57±9.22	0.731 (df=78)	0.467
12^th^ month	16.44±10.70	15.44±9.79	0.446 (df=82)	0.016*

* P<0.05

## Discussion

Published research in the area of alcoholism treatment outcome emerges predominantly from the United States where strong community follow-up facilities exist.

In a chronic disease like alcoholism, where outcomes are comparable with other chronic medical illnesses,(8) the need for continued care cannot be overstated. The findings of Project Match,(3) which compared three different time-limited therapies for alcohol dependence, showed that although there were significant improvements in all groups, many patients returned to alcohol use following treatment. This finding also underscores the need for continuing care. It has been suggested that social and community resources that are available for long periods of time are more likely to have a lasting influence on the course of alcoholism.(5)

This study examined the role of providing continued care and vocational rehabilitation in a group of alcohol dependent individuals as compared with age-matched controls, both groups having received similar, initially intensive hospital-based treatment for alcohol dependence. Our findings suggest that monitored ‘after care’ sustains the improvements made by alcohol dependents during their in-patient treatment. It has been suggested that brief contacts, both actual follow-up(11) and even telephonic contacts,(12) have the ability to support abstinence and prevent relapses. Our intervention consisted mainly of ongoing personal contact with treating staff, support for person and family, as well as supported employment. Supported employment allows the person to develop good work habits and also allows more frequent contact with counsellors to reinforce recovery and non drug use. Although affiliation with groups like the Alcoholics Anonymous has been shown to improve outcome,(13) attempts to refer to groups such as AA did not work in our study group.

The study group was identified from a single geographically determined area, a slum. This was because the study emerged from a community-based drug rehabilitation project. Addiction treatment services for marginalized groups living in slums are woefully inadequate particularly in developing countries like India, and for the few who had previously accessed treatment services at the hospital, early relapse had been a rule. The improvements sustained in this project were, therefore, singularly significant. One of the limitations of the study is the ratings on the alcohol drinking that were made at each 3-month follow-up visit, for the prior month alone. Although this may not be reflective of the entire 3-month period, it was done to overcome the problem of retrospective recall.

Despite the limitations of the study (small sample size, study group drawn from a defined slum area), we have demonstrated, in a prospective design, the sustaining effect of continued care in alcohol dependence. This study emphasizes the need for a more comprehensive and continuing care approach to maintain gains of initial interventions.
